# Migraine among medical students in Kuwait University

**DOI:** 10.1186/1129-2377-15-26

**Published:** 2014-05-10

**Authors:** Jasem Y Al-Hashel, Samar Farouk Ahmed, Raed Alroughani, Peter J Goadsby

**Affiliations:** 1 Department of Neurology, Ibn Sina Hospital, P.O. Box 25427, Safat 13115, Kuwait City, Kuwait; 2 Department of Medicine, Faculty of Medicine, Health Sciences Centre, Kuwait University, Kuwait City, Kuwait; 3 Department of Neurology and Psychiatry, Al-Minia University, Minia, Egypt; 4 Division of Neurology, Amiri Hospital, P.O. Box 1661, Qurtoba 73767, Kuwait City, Kuwait; 5 Division of Neurology, Dasman Diabetes Institute, P.O. Box 1180, Dasman, 15462, Kuwait City, Kuwait; 6 NIHR-Wellcome Trust Clinical Research Facility, King’s College London, London, UK

**Keywords:** Migraine, Prevalence, Medical students, Kuwait

## Abstract

**Background:**

Medical students routinely have triggers, notably stress and irregular sleep, which are typically associated with migraine. We hypothesized that they may be at higher risk to manifest migraine. We aimed to determine the prevalence of migraine among medical students in Kuwait University.

**Methods:**

This is cross-sectional, questionnaire-based study. Participants who had two or more headaches in the last 3 months were subjected to two preliminary questions and participants with at least one positive response were asked to perform the validated Identification of Migraine (ID Migraine™) test. Frequency of headache per month and its severity were also reported.

**Results:**

Migraine headache was suggested in 27.9% subjects based on ID-Migraine™. Migraine prevalence (35.5% and 44%, versus 31.1%, 25%, 21.1%, 14.8%, 26.5%, *p* < 0.000), frequency (5.55 + 1.34 and 7.23 + 1.27, versus 3.77 ± 0.99, 2.88 ± 0.85, 3.07 ± 0.96, 2.75 ± 0.75, 4.06 ± 1.66, p < 0.000); and severity of headache (59.1% and 68.2%, versus 28.3%,8.3%, 6.7%,16.7%, p < 0.000; were significantly increased among students in the last 2 years compared to first five years of their study. Stress 43 (24.9%), irregular sleep 36 (20.8%), and substantial reading tasks 32 (18.5%), were the most common triggering factors cited by the students.

**Conclusion:**

The prevalence of migraine is higher among medical students in Kuwait University compared to other published studies. The migraine prevalence, frequency and headache severity, all increased in the final two years of education.

## Background

Migraine is a neurological disorder that represents a significant global health problem due to its frequency and substantial disability [1]. Migraine has a prevalence of 12–18%, which has been shown to be both age and gender dependent in community-based studies worldwide [2].

Migraine is highly prevalent among university students and it is associated with impaired academic performance and limited daily activities [3]. It has negative effects among university students, who indeed require constant concentration and high level of performance [4]. Students with migraine type headaches missed more school than other students [5]. Among university students, decreased school performance limits success, this may influence their future occupational performance in the society [6].

Although numerous studies have assessed the prevalence of migraine within the general population, professional groups, and industrial/work place settings, there were few studies focused on the prevalence of migraine in university students [7-11]. The prevalence of migraine among medical students ranges from 11 to 40% worldwide [1,12,13]. The epidemiology of migraine among medical students is of a particular interest since migraine is the most common type of headache in young adults [6] and its frequency has been increased during students educational years of study [14].

Previous studies showed that stress, sleeplessness, eating habits, menstrual cycle, changes in weather conditions and temperature, frequent traveling, food items, oral contraceptives and physical activities are factors that most trigger migraine attacks. The most common migraine triggers are lack of sleep and fatigue along with smoking that all can be a precipitating factor for migraine attacks [15-17].

As medical students are subjected to stress regarding their exams, high level performance, many years of education and the implicit responsibility to the courses, we aimed to determine the prevalence of migraine headache among our medical students in Kuwait University using the quick screening tool ID Migraine™ test [18].

## Methods

A cross-sectional, questionnaire-based study was conducted on students of the faculty of Medicine, Kuwait University. In 2012-2013 academic year, 808 students registered, and we were able to reach only 640 (79.8%) students who filled out the questionnaire versus 168 (20.7%) non responders. Participants were informed about the study and their verbal consent was obtained. After the data collected, 621(76.9%) participants were included in the study, the rest We added the percentage of responders 640 (79.2%) versus non responders 168 (20.7%). The included participant were 621 (76.9%). The other 19 (2.4%) participant were excluded because of their incomplete questionnaires.

The data for this study was collected from the students’ self-reports by filling the questionnaires. Questionnaire data included demographic information (age, sex), stepwise evaluation to determine the prevalence of migraine and clinical characteristics of the headache, i.e. frequency per month, intensity [19], and triggering factors.

### Initial evaluation

A stepwise evaluation was used to determine the prevalence of migraine type headache. Students who replied “yes” to the first question: “Did you have two or more headaches in the last 3 months?”, were considered the subjects with headache that was potentially troublesome. They were then asked: “Do you have headaches that limit your ability to study or enjoy life?”, and “Do you want to talk to your healthcare professional about your headaches?”. Participants who gave one positive response out of two, were considered as the subjects who are likely to have migraine type headaches and asked the three item ID Migraine™ test [18].

### ID-migraine

The final three-item screening questions of the ID Migraine™ test are: During the last 3 months, 1. Did you feel nauseated or sick in your stomach with your headaches? 2. Did light bother you when you had a headache (a lot more than when you do not have headaches)? 3. Did your headache limit your ability to work, study or do what you needed to do for at least 1 day? A test-diagnosis of migraine headache required at least two positive responses.

Identification of Migraine (ID Migraine™) is widely used as a screening instrument for identifying migraineurs at primary health services. It has a three questions (items) screening tool for migraines that has demonstrated good validity [20]. Each of the three items relates to a central diagnostic symptom of migraine: nausea, photophobia, and interference with activities. Each question is scored dichotomously with endorsements of two or more items suggesting probable migraine. Sensitivity, specificity and positive predictive value of this test in primary care have been defined as 81%, 75% and 93%, respectively [20]. The test is also validated to be used among adolescent students with a sensitivity of 62.1% and specificity of 71.1% [21].

### Headache severity

Headache pain intensity was measured on a four-point scale where 0 = no headache; 1 = mild headache; 2 = moderate headache; 3 = severe headache. This scale is recommended for use in migraine research by the International Headache Society [19].

### Triggers

Students were asked also to indicate triggers for their headaches. A list of common triggers was provided in the questionnaire and included the following items: exposure to sun, emotional stress or anxiety, noise, exams, reading hours, eating habits, fasting, menstruation, irregular sleep, physical activity, and smoking.

### Data analysis

Collected data was analyzed to assess the prevalence, frequency, severity and triggering factors of headache. Statistical Package for the Social Sciences (SPSS) for Windows version 20 was used. Simple descriptive statistical tests (Mean and Standard deviation) are used to describe the numerical values of the sample and frequency described, and the number and percentage of the non-numerical values. A comparison of variables between males and females and was performed using the Independent *t* test for numeric variables and the chi-square (*χ*^2^) test for non-numeric variables. The significance of the differences of mean frequency of headache between faculty grades were compared by using the one way ANOVA test. A comparison of prevalence of headache and pain intensity between faculty grades was performed using the chi-square (*χ*^2^). A *P* < 0.05 was regarded as significant.

## Results

Out of 808 registrants, 621 students were included in the study. A total of 184 (29.6%) were males and 437 (70.4%) were females. The females are more frequent in all grades (71.5%, 63.5%, 59.2%, 57.3%, 73.%, 69.4%, 84%). They are more frequent if seventh grade followed by the first grade and sixth grade.

### ID-migraine

The prevalence of migraine among medical students according to ID Migraine™ was 27.9%. Thirty-seven were male (20.1%) and 136 were female (31.1%). Table 1 displays the demographic and clinical characteristics of headache in students with migraine in our study. The mean age and age of onset of the migraine among students were 20 ± 2 and 16 ± 7. years, respectively. Migraine was significantly more prevalent among females compared to males. There was no significant difference between males and females as regard age, age of onset, duration of the migraine, frequency and the severity of the headache.

**Table 1:** Demographic and clinical characteristics of headache in migraine students

**Variables**	**Total (n = 173)**	**Males (n = 37)**	**Females (n = 136)**	***P***
**Prevalence of migraine**	173/621	37/184	136/437	0.02*
**N (%)**	(27.9%)	(20.1%)	(31.1%)	
**Age in years (M** ± **SD)**	20.17 ± 2.29	20.14 ± 2.38	20.18 ± 2.27	0.91
**Range**	16-25	17-24	16-25	
**Age at onset in years (M + SD)**	16.70 ± 2.12	16.41 ± 2.05	16.79 ± 2.14	0.34
**Range**	12-23	13-23	12-23	
**Duration of migraine in years (M + SD)**	3.48 ± 1.89	3.73 ± 2.34	3.41 ± 1.75	0.36
**Range**	1-9	1-9	1-8	
**Frequency of headache per month (M + SD)**	4.29 ± 1.80	4.16 ± 2.01	4.32 ± 1.74	0.63
**Range**	2-9	2-8	2-9	
**Severity of headache**				
**Mild**	49 (28.3%)	9 (24.3%)	40 (29.4%)	0.43
**Moderate**	82 (47.4%)	22 (59.5%)	60 (44.1%)	
**Severe**	42 (24.3%)	6 (16.2%)	36 (26.5%)	

*=Significant.

### Distribution by grade in faculty of medicine

Out of 193 students in grade one, 60 were found to have migraine (31.1%), 22 out of 62 (35.5%) in the sixth grade and 22 out of 50 (44%) in the last grade. Migraine prevalence was found to be significantly higher in last 2 grades and first grade compared to other grades (*p* < 0.000). However, the frequency per month and the severity of migraine headaches were significantly increased during the last two grades only, (6 ± 1.34 and 7 ± 1.27, p < 0.000; 6 ± 0.76 and 7 ± 1.25, p < 0.000), respectively (Table 2).

**Table 2:** Comparison of migraine prevalence, frequency and severity among grades of medical students

**Variables**	**Grade 1 (n = 60)**	**Grade 2 (n = 24)**	**Grade 3 (n = 15)**	**Grade 4 (n = 12)**	**Grade 5 (n = 18)**	**Grade 6 (n = 22)**	**Grade 7 (n = 22)**	**P**
**Prevalence No (%)**	60/193 (31.1%)	24/96 (25%)	15/71 (21.1%)	12/81 (14.8%)	18/68 (26.5%)	22/62 (35.5%)	22/50 (44%)	0.000**
**Frequency of headache (per month)**	3.77 ± 0.99	2.88 ± 0.85	3.07 ± 0.96	2.75 ± 0.75	4.06 ± 1.66	4.95 ± 1.76	6.00 ± 2.18	0.000**
**Range**	2-6	2-5	2-5	2-4	2 –7	2- 8	3-9	
**Severity of headache**								
**Mild**	9 (15%)	11 (45.8%)	10 (66.7%)	8 (66.7%)	10 (55.6%)	1 (4.5%)	1 (4.5%)	0.000**
**Moderate**	34 (57%)	11 (45.8%)	4 (26.7%)	4 (33.3%)	5 (27.8%)	8 (36.4%)	6 (27.3%)	
**Severe**	17 (28.3%)	2 (8.3%)	1(6.7%)	0	3 (16.7%)	13 (59.1%)	15 (68.2%)	

**= Highly significant.

### Triggers

Stress 43 (24.9%), irregular sleep 36 (20.8%), and substantial reading 32 (18.5%) were the most common triggers for headache followed by exams 19 (11.1%), smoking 10 (5.8%) and fasting 10 (5.8%; Table 3).

**Table 3:** Trigger factors of headache among migraine students (n = 173)

**Migraine triggers**	**No (%)**
**Stress**	43 (24.9%)
**Irregular sleep**	36 (20.8%)
**Much Reading**	32 (18.5%)
**Exams**	19 (11.1%)
**Smoking**	10 (5.8%)
**Fasting**	10 (5.8%)
**Eating habits**	7 (4%)
**Physical activity**	6 (3.4%)
**Exposure to sun**	6 (3.4%)
**Menstruation in females**	4 (2.3%)

## Discussion

The present study was conducted among medical students registered to the faculty of medicine, Kuwait University in the academic year of 2012 – 2013. To date, this study is the first university survey evaluating the prevalence of migraine based on self-report in Kuwait University.

The Medical School curriculum at Kuwait University requires constant concentration, continuous effort and hard work, even one day absence from studies can affect the student’s school performance and success. Evaluating and managing headaches among medical students becomes especially important for students themselves and for us as treating physicians.

Migraine prevalence based on ID Migraine™ among medical students in Kuwait University was 27.9% (21.4% in males versus 31.1% in females). The prevalence of migraine among medical students is variable worldwide. We found that the prevalence of migraine in our cohort is higher compared to other international studies. The prevalence of migraine among medical students reported to be 33.8% in Nairobi [13], 22% in Brazil [12], 14.1% in Nigeria [10], 13.1% in South East Nigeria [22], 12.6% in Turkey [6], 12.2% in in Oman [11] and 7.14% in the Southeast of Iran [23]. Most of our students were females 70.4% which could explain the higher frequency of migraine in our study compared to others. The difference in migraine prevalence between our study and other studies could be explained also by the methodological differences or different self-reporting questionnaires used and the time of the study which differs with various studies. A study conducted in a stressful period, such as midterms or final examinations, would likely reveal a higher prevalence of migraine headache as the screening test is based on the presence of headache in the last three months as in our study. The difference also may be attributed to racial, environmental, different socioeconomic status, climate, or nutritional habits which could be contributing factors for migraine in these different countries [24].

The migraine prevalence in medical students in our cohort is higher compared to migraine prevalence (not medical students) in Arab Gulf countries: 2.6–5% in Saudi Arabia [25,26] and 7.9% in Qatar [27] and the one-year migraine prevalence was 10.1% in Oman [28]. These countries are somehow similar culturally, economically, climatically and ethnically to Kuwait. The difference between our results and that of Arab Gulf countries may be due to the methodological differences that influence the results of epidemiological studies in headache [29]. The age of the studied population varied across these studies, some being inclusive of all adults and children, while others included only adults. Our study included only adolescents and young adults. Application of screening questions is also relevant, as differences would be expected when applying key questions such as, “Have you ever suffered from headache?” versus “Have you had a headache within the past year?”, or “past three months”, as in our study [30].

In our study, migraine prevalence was 1.5 times more common in females than males which are in agreement with the literature and previous studies [10,14,24]. There was no significant difference between males and females with regard to age of onset and character of the headache. This is likely explained by similar triggers exposure for both genders in our study are stress, irregular sleep, reading hours and exams.

Our migraine students had experienced 4.2 attacks per month and their mean pain intensity was 5.6 which is in line with previous studies. The mean attack frequency per month was 3.8 among University Students in Cotonou University, Benin [31], 4.48 among university students in Bigal study, Brazil [5] and 5 attacks per month among medical students in Turkey [8]. Most, migraine attacks were moderate to severe in the study of Ezeala-Adikai and his colleagues in Nigeria [22].

The prevalence of migraine in our study was found to be highest among seventh grade (the last grade) followed after by the sixth grade. The high frequency rate of migraine among the last two grades is most likely explained by the high frequency of exams which are associated with increasing stress, irregular sleep pattern, many reading hours and fasting; all were triggers of headache in our study. The greater prevalence of migraine among the last grade compared with the previous grades is consistent with the study of Galinović and his colleagues [14].

Surprisingly, migraine prevalence was also high in the first grade. Students of the first grade in the faculty of medicine, Kuwait University, showed an interesting finding with high frequency of migraine. Likely this high prevalence is secondary to the emotional stress this group of students face in this year since by the end of the academic year only 40-50% of both males and females will be eligible to continue in the faculty of medicine, while the rest will be distributed to the faculty of pharmacy and dentistry, all of which will depend on their final score. This seems a plausible explanation for their high migraine frequency.

We predefined list of triggers in our headache questionnaire. Some students have more than one trigger factor, but we calculated the most trigger factor for each one. Although, menses was a trigger factor among 20% of our female cohort but the irregular sleeping and much reading were more precipitating of migraine attacks. In the present study, emotional stress or anxiety, irregular sleep, reading hours, exams, smoking and fasting were the most common trigger of migraines. We are in agreement with the results of previous studies who reported that stress is the commonest triggering factor for migraine headache in medical students [1,22]. Our results are also consistent with Timothy and his colleagues [32], who concluded that migraine can be triggered by exams, diet, hunger, sleep deprivation, physical and emotional stress.

## Limitation of the study

The migraine students were not interviewed by neurologist for confirmation of migraine diagnosis. In addition, the use of a self-administered questionnaire might create a misunderstanding of some asked questions with the risk of subjectivity in the answers.

## Conclusions

Migraine prevalence among medical students in Kuwait University is high compared to other studies. It was significantly higher in females. Prevalence, frequency and severity of headache increase with years of educations. Stress, irregular sleep, and reading hours were the most common triggering factors.

## Recommendation

As migraine is highly prevalent among medical students, primary care physicians should be aware of its prevalence among those students for early diagnosis and management. Accurate early diagnosis of migraine and adequate management can be beneficial for students to enhance their academic performance.

## Competing interests

The authors have no financial obligations to disclose related to this study.

## Authors’ contributions

J Y designed the study and carried out the data collection. SF participated in the design of the study, performed the statistical analysis and drafted the manuscript. R drafted the manuscript. PJG reviewed the manuscript. All authors read and approved the final manuscript.
